# Spinal movement variability associated with low back pain: A scoping review

**DOI:** 10.1371/journal.pone.0252141

**Published:** 2021-05-24

**Authors:** Hiroki Saito, Yoshiteru Watanabe, Toshiki Kutsuna, Toshihiro Futohashi, Yasuaki Kusumoto, Hiroki Chiba, Masayoshi Kubo, Hiroshi Takasaki

**Affiliations:** 1 Department of Physical Therapy, Tokyo University of Technology, Ota-ku, Tokyo, Japan; 2 Department of Physical Therapy, Secomedic Hospital, Funabashi, Chiba, Japan; 3 Postgraduate School, Saitama Prefectural University, Koshigaya, Saitama, Japan; 4 Department of Physical Therapy, Niigata University of Health and Welfare, Niigata, Niigata, Japan; 5 Department of Physical Therapy, Saitama Prefectural University, Koshigaya, Saitama, Japan; University of Maryland School of Medicine, UNITED STATES

## Abstract

**Objective:**

To identify suggestions for future research on spinal movement variability (SMV) in individuals with low back pain (LBP) by investigating (1) the methodologies and statistical tools used to assess SMV; (2) characteristics that influence the direction of change in SMV; (3) the methodological quality and potential biases in the published studies; and (4) strategies for optimizing SMV in LBP patients.

**Methods:**

We searched literature databases (CENTRAL, Medline, PubMed, Embase, and CINAHL) and comprehensively reviewed the relevant papers up to 5 May 2020. Eligibility criteria included studies investigating SMV in LBP subjects by measuring trunk angle using motion capture devices during voluntary repeated trunk movements in any plane. The Newcastle-Ottawa risk of bias tool was used for data quality assessment. Results were reported in accordance with the Preferred Reporting Items for Systematic Reviews and Meta-Analyses Extension for Scoping Reviews.

**Results:**

Eighteen studies were included: 14 cross-sectional and 4 prospective studies. Seven linear and non-linear statistical tools were used. Common movement tasks included trunk forward bending and backward return, and object lifting. Study results on SMV changes associated with LBP were inconsistent. Two of the three interventional studies reported changes in SMV, one of which was a randomized controlled trial (RCT) involving neuromuscular exercise interventions. Many studies did not account for the potential risk of selection bias in the LBP population.

**Conclusion:**

Designers of future studies should recognize that each of the two types of statistical tools assesses functionally different aspects of SMV. Future studies should also consider dividing participants into subgroups according to LBP characteristics, as three potential subgroups with different SMV characteristics were proposed in our study. Different task demands also produced different effects. We found preliminary evidence in a RCT that neuromuscular exercises could modify SMV, suggesting a rationale for well-designed RCTs involving neuromuscular exercise interventions in future studies.

## Introduction

Low back pain (LBP) has a high recurrence rate, and its symptoms are often prolonged [1]. A possible physical reason for the recurrent and prolonged nature of LBP includes altered spinal motor control [2, 3]. Spinal movement variability (SMV) represents one of the measures for altered spinal motor control in LBP patients [4, 5] and is defined as the natural variations of trunk movement relative to adjacent segments such as the pelvis [6–8]. While a certain range of SMV plays an important role in maintaining healthy tissue states, an excessive degree of SMV is thought to be a potential risk factor for LBP [8]. Several studies indicated that excessively decreased SMV results in the concentration of load on certain tissues of the trunk, which can inhibit tissue remodeling and result in tissue damage [9, 10]. Likewise, excessively increased SMV leads to inconsistent or unstable spinal movement, which can increase tissue strains of the trunk [11, 12].

In studies investigating SMV in individuals with LBP, there are several methodological considerations that need to be addressed. First, a diverse range of methodologies and statistical tools for the assessment of SMV in LBP patients currently exist in the literature [10, 13, 14]. Broadly, SMV can be measured by linear and non-linear statistical tools [15]. Linear statistical tools reflect the amount of variability and quantify the magnitude of variation in movement [15]. The metric includes standard deviation, range and root mean square [8, 16, 17]. Non-linear statistical tools consider additional information regarding time-dependent structures of the system embedded in a movement sequence [15]. Thus, non-linear statistical tools quantify the structure of variability, and allow us to understand the adaptability of a biological system towards changing conditions [15]. As each tool has a different aspect of variability, data synthesis is challenging. Therefore, it is considered necessary to comprehensively understand the available methodologies and statistical tools in assessing SMV in LBP patients in order to determine the optimal methodological designs of future studies. Secondly, there seems to be inconsistent findings in the SMV associated with LBP across cross-sectional studies [16, 18, 19]. This is not surprising given the heterogeneous nature of LBP [20]. To develop diagnostic classifications that guide the management strategies for LBP, it would be essential to identify the subgroups of LBP patients [20]. Therefore, it is important to elucidate the potential subgroups of LBP based on SMV, and clarify the influence of different methodological approaches such as selection of LBP and movement tasks on the identification of subgroups. Lastly, there seems to be a diverse range of interventions and inconsistent findings on the changes in SMV in prospective studies [21–23]. Comprehensive understanding of the possible interventions for LBP patients would be beneficial for designing high quality clinical trials [24].

Therefore, it was considered prudent to undertake a scoping review, which is a suitable method for addressing an exploratory research question that allows for the mapping of key concepts, the different types of evidence, and the gaps in a research area [25]. The primary goal of this systematic scoping review was to identify suggestions for future research by undertaking the following four steps: (1) investigation of the methodologies and statistical tools used in assessing SMV in LBP patients; (2) identification of characteristics that influence the direction of change in the SMV associated with LBP in cross-sectional studies; (3) evaluation of the methodological quality and potential biases in the published studies; and (4) identification of strategies for optimizing SMV in LBP patients in prospective studies.

## Materials and methods

### Study design and methodology

This scoping review was conducted according to the framework originally developed by Arksey and O’Malley [26] and recently modified by several other authors [25, 27, 28]. The review was conducted according to the following steps: (1) identification of the research question; (2) identification of relevant studies; (3) selection of relevant studies; (4) charting of data; and (5) collation, summarisation, and reporting of results [25, 27, 28]. Results were reported in accordance with the Preferred Reporting Items for Systematic Reviews and Meta-Analyses Extension for Scoping Reviews (PRISMA-ScR) [29].

### Identification and selection of relevant studies

The inclusion and exclusion criteria are presented in Table 1. The following databases were searched by the first author (HS): CENTRAL, Medline, PubMed, Embase, and CINAHL. Of note, as searches of Medline and PubMed did not retrieve the same records [30], PubMed was included in addition to Medline to improve our search strategy. Searches covered articles published between database inception and 5 May 2020. S1 File presents our search strategy on Medline. In addition to database searches, we manually searched relevant reviews of kinematic changes associated with LBP [31–35], as well as focused reviews of movement variability [4, 10, 15, 20].

**Table 1:** Eligibility criteria for inclusion and exclusion.

Inclusion criteria	Exclusion criteria
1) Primary studies that assessed intra-individual SMV in LBP patients using linear or non-linear statistical tools	1) Studies that included LBP patients with specific spinal pathologies (e.g., scoliosis, spinal stenosis, spondylolisthesis, degeneration, disc herniation), a history of surgical management, serious pathology (e.g., fracture, infection, cancer, central nervous system disease, or respiration disorders), those who were pregnant, and those with a history of childbirth within 3 months
2) Studies that measured the trunk angle relative to the pelvis or thigh using motion capture devices during voluntary repeated trunk movements in any plane (e.g., forward bends, rotation, lifting)	2) Secondary studies (e.g., systematic review, overview, narrative reviews, integrative reviews)
3) English language and peer-reviewed publications	3) Inclusion of children and adolescents (< 18 years of age), and cadaveric studies

Before the screening process, each assessor received background information about the review topic and a table of inclusion and exclusion criteria [36]. Because the number of screening abstracts was relatively large, calibration exercises were performed to ensure consistency throughout the process [29, 37]. Thus, assessors started with a pilot phase to screen for relevant publications based on the eligibility criteria, followed by a discussion on how the inclusion criteria should be applied [29, 37]. The literature was then equally divided into three parts, each of which was screened by one of three pairs of assessors (HS and YK, HC and TF, YW and TK) through title and abstract review, without being blinded to the authors. Assessors were instructed to include the abstracts if there was insufficient information for a definitive decision [36]. Two reviewers (HS and YK) then performed the full-text inspection. Members of each pair initially conducted the screening and full-text inspection processes independently of each other. Disagreements between assessors regarding the selection of studies were then resolved by consensus and discussion [38].

### Assessment of study quality

Although several methodological quality assessment tools currently exist, a gold-standard has not been established [39]. The Newcastle-Ottawa Scale (NOS) is one of the tools frequently used in systematic reviews [31, 40, 41]. The NOS evaluates the definitions of cases and controls independently, and assesses whether recruited participants are representative of the target population, and whether the control series used in the study is derived from the same population as the cases [42]. Given the heterogeneity of the LBP population who showed different motor control strategies for LBP [20], we thought these aspects were important because different inclusion criteria of LBP may impact SMV. Furthermore, designing an adequate control group with evidence of no history of LBP is also necessary to investigate the differences in SMV between LBP patients and controls. Hence, the purpose of exploring the methodological quality of the included studies using NOS was to investigate the influence of these methodological differences on SMV results (i.e., difference in SMV between LBP patients and controls, changes in SMV over time with or without intervention).

The NOS consists of eight items that assess three elements of methodological quality: group selection (four items, 0–4 points), group comparability (one item, 0–2 points), and ascertainment of outcome (three items, 0–3 points) [42, 43]. A score of ≥ 8 was considered high quality [31].

Two reviewers (HS and YW) independently appraised the included studies using the NOS [42]. Of note, we used a modified version of the NOS, which was adapted to LBP [43]. S2 File presents the details of the quality assessment score. Disagreements were resolved by discussion. The level of initial agreement between the two reviewers was examined by calculating Cohen’s kappa (κ) and percent agreement. The κ values were interpreted as follows: ≤ 0.40, poor agreement; 0.41–0.60, moderate agreement; 0.61–0.80, good agreement; 0.81–1.00, very good agreement [44].

### Data extraction

Data extraction was conducted by two independent reviewers (HS and YK) according to the Population, Exposure, Comparator, Outcomes, Study Design (PECOS) framework. Any disagreement was resolved through discussion. Parameters included study population characteristics (inclusion criteria for LBP and control groups, number of participants, participant age and gender), sample source, LBP symptom duration, pain or disability scale, and psychological characteristics such as the Pain Catastrophizing Scale [45] and the Tampa Scale for Kinesiophobia [46] used to measure LBP. Exposure parameters included movement tasks that measured SMV, which could involve repeated spinal movements of any plane (e.g., forward bends, rotation, lifting). Comparator parameters included non-LBP or LBP individuals with different characteristics. Outcome parameters included differences in outcome between LBP and control groups, metrics used to analyze the SMV. As such, two types of statistical tools to measure SMV were extracted: the linear statistical tools and non-linear statistical tools [15, 47]. Reliability of measurement tools used or referenced in each study were also recorded. Study design parameters included cross-sectional and prospective studies (cohort and experimental studies).

### Synthesis

All studies were classified according to study design, LBP characteristics including chronicity, intensity, psychological characteristics, the direction of change in SMV, and statistical analysis methods used to assess SMV. The main results of each study were also summarized.

## Results

### Study selection

**Fig 1:**
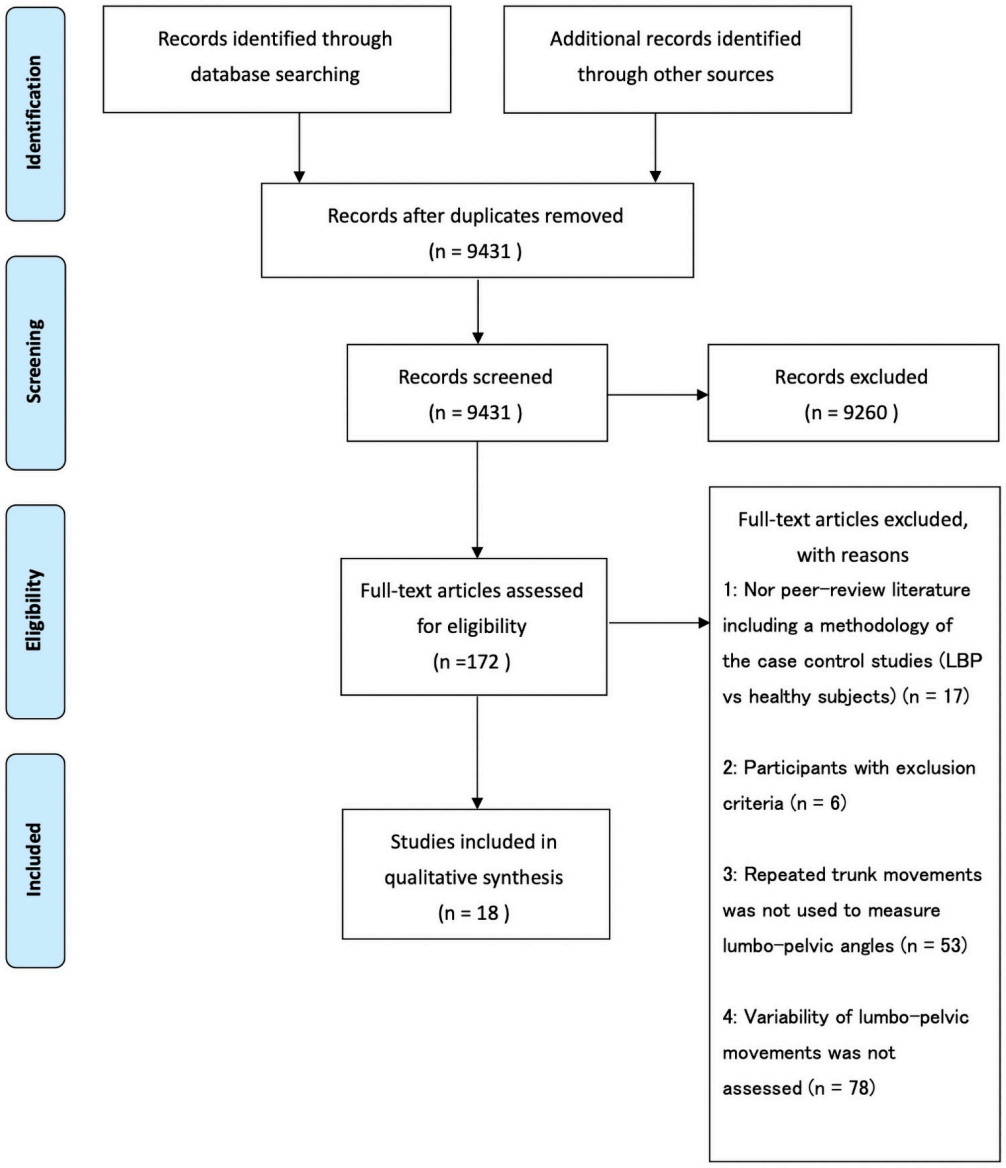
Flowchart of study selection process.

Fig 1 presents the flowchart of the study selection process. After full text inspection, 154 studies were excluded, with reasons summarized in the S1 Table, while 18 studies [6, 8, 16–19, 21–23, 48–56] were chosen for data extraction. The rate of agreement with regard to study inclusion was 98.5% during initial screening and 85.3% during the full text inspection.

### Study characteristics

Of the 18 studies included in the review, 78% were cross-sectional in design [6, 8, 16–19, 48–54, 56], the vast majority of which (72% of total studies) individuals with and without LBP. The remaining 22% were prospective studies [21–23, 55], including a prospective cohort study [55] and three interventional studies [21–23]. With regard to LBP chronicity, 78% of studies examined chronic LBP [6, 8, 16, 19, 48–52, 56], 39% examined sub-acute LBP [6, 22, 49, 50, 53, 55, 56], 22% examined acute LBP [21, 53–55]. In addition, 14% examined mechanical LBP [18, 54, 57]. S2 Table presents the key methodological details of each study (e.g., eligibility criteria, and measurement apparatus).

### Methodological considerations

Table 2 presents the NOS scores of each study. The κ value for agreement between the NOS scores given by each reviewer was 0.75 (95% confidence interval 0.66–0.85), indicating good agreement. The percentage agreement was 84.0%.

**Table 2:** Summary of the Newcastle-Ottawa Scale (NOS) for each study.

		Selection				Comparability	Exposure			Total Score
Study	Year	1 adequate case definition	2 representativeness of the cases	3 selection of controls	4 definition of controls	5 comparability of cases and controls	6 ascertainment of exposure	7 same method of ascertainment for cases and controls	8 reporting of non-response rate	
Adamantios [23]	2017	1	0	0	0	2	0	1	1	5
Asgari [16]	2015	1	0	N/A	1	0	0	1	N/A	3
Asgari [48]	2017	1	0	1	1	2	0	1	N/A	6
Bauer [56]	2015	0	0	0	0	0	0	1	1	2
Bauer [6]	2017	0	0	0	0	0	0	1	1	2
Bauer [22]	2019	0	0	1	0	2	0	1	1	5
Chehrehrazi [17]	2017	0	0	1	0	2	0	1	N/A	4
Dideriksen [19]	2014	0	0	0	1	2	0	1	N/A	4
Graham [49]	2014	1	0	1	0	2	0	1	N/A	5
Ippersiel [50]	2018	0	0	0	N/A	0	0	1	N/A	1
Mokhtarinia [8]	2016	1	0	1	0	2	0	1	1	6
Moreno [51]	2018	0	0	N/A	0	0	0	1	1	2
Pranata [52]	2018	1	1	1	1	1	0	1	N/A	6
Shojaei [53]	2017	0	0	0	1	N/A	0	1	N/A	2
Shojaei [55]	2019	1	0	0	0	0	0	1	N/A	2
Silfies [18]	2009	1	1	0	N/A	0	0	1	N/A	3
Williams [54]	2013	1	0	1	0	0	0	1	N/A	3
Williams [21]	2014	1	0	0	0	0	0	1	N/A	2

All studies except three [8, 48, 52] received NOS scores less than 6/9. Most notably, relatively few studies received scores for more than one of the four NOS items assessing group selection: adequate case definition was present in 55.5%, appropriate selection of controls in 38.8%, appropriate definition of controls in 27.7%, and good representativeness of cases in 11.1%.

### Methodologies used to assess SMV in LBP patients

**Fig 2:**
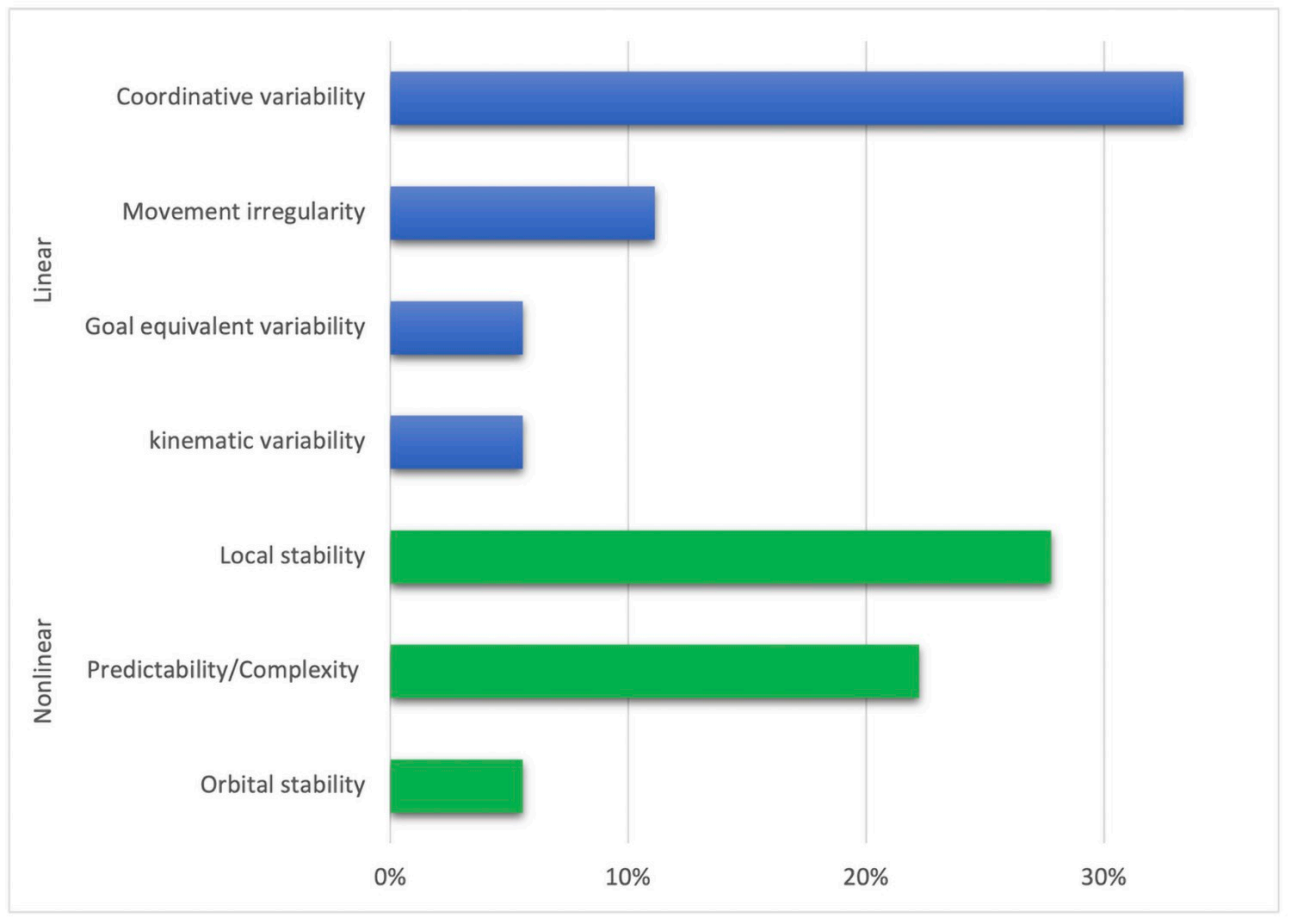
The frequency in the type of spinal movement variability (SMV) across the studies.

Fig 2 presents the frequency in the type of SMV across the studies. Among the ten studies [8, 16–18, 21, 50, 52–55] that employed linear statistical methods, Coordinative variability was most commonly assessed using the continuous relative phase (CRP) curve [8, 18, 50, 52, 53]. Eight studies [6, 16, 19, 22, 48, 49, 51, 56] used non-linear statistical methods, where Lyapunov exponent, as a measure of local stability, was most frequently used [16, 23, 48, 49, 51]. Frequently used movement tasks included trunk forward bending and backward return [6, 8, 16–18, 21, 49, 53–55] and object lifting [19, 21, 22, 48, 51, 52, 54, 56].

### The SMV with and without LBP

Table 3 presents a summary of cross-sectional studies that assessed the differences in SMV between individuals with and without LBP, as well as those within the LBP population [6, 8, 16–19, 48–54, 56]. Six cross-sectional studies [6, 8, 16, 19, 53, 54] reported decreased SMV in LBP patients. Of these, three studies [6, 53, 54] examined acute and/or sub-acute LBP, while one [54] involved moderate LBP intensity. In addition, one study [19] found that LBP associated with mild-to-moderate fear of movement. Four cross-sectional studies [18, 50, 54, 56] found greater SMV in individuals with LBP compared with those without, and LBP in these studies was generally chronic and/or mechanical. Five cross-sectional studies [17, 48, 49, 51, 52] reported no difference in SMV between individuals with and without LBP. LBP participants in these studies generally had sub-acute or chronic LBP [17, 48, 49, 51, 52] with those in one study reporting relatively lower pain intensity (visual analogue scale score <2/10) [17], and those in another having low levels of disability (mean scores of 4.0 on the Roland-Morris Disability Questionnaire and 7.8 on the Oswestry Disability Index) [49].

**Table 3:** Summary of cross-sectional studies that assessed spinal movement variability (SMV) in individuals with low back pain (LBP).

Study	Study Design	LBP characteristics				Task performance	Types of SMV	Statistical tool	Result
		Chronicity	Pain/disability level	Psychological factor	Others				
Decreased SMV									
Williams (2013)	Cross-sectional (Acute LBP vs Chronic LBP)	Acute	VAS: Acute LBP: 6.2 ± 1.9	TSK: Acute LBP: 39.3 ± 4.1	Mechanical	Forward and backward return, side-bending, twisting, and object lifting	Movement irregularity	Angular velocity-ROM plots	Decreased SMV during most tasks in acute LBP compared to chronic LBP
			Chronic LBP: 4.6 ± 2.2	Chronic LBP: 38.3 ± 7.5					
Shojael (2017)	Cross-sectional (LBP vs Healthy)	Acute/sub-acute	—	—	—	Forward bending and backward return	Coordinative variability	CRP curve	Decreased SMV in LBP
Mokhtarinia (2016)	Cross-sectional (LBP vs Healthy)	Chronic	VAS < 2	—	—	Forward bending and backward return	Coordinative variability	CRP curve	Decreased SMV when both symmetry and velocity were highly demanding in LBP
Bauer (2017)	Cross sectional (LBP vs Healthy)	Sub-acute/chronic	NRS ≥ 1 ODI > 8	—	STarT Back < 4	Forward bending and backward return (sitting)	Predictability and/or Complexity	Determinism, Sample entropy	Decreased SMV in the presence of fatigue in LBP
Dideriksen (2014)	Cross-sectional (LBP vs Healthy)	Chronic	NRS: 3.1 (2.2) ODI: 14.2 (7.2)	TSK: 31.8 (5.9) PCS: 16.1 (8.5) STAI: 40.2 (7.1)	SF-36 (total): 66.9 (12.2)	Object lifting	Predictability and/or Complexity	Determinism	Decreased SMV in LBP
					SF-36 (physical): 60.9 (14.2)				
					SF-36 (mental): 67.6 (14.1)				
Asgari (2015)	Cross-sectional (LBP vs Healthy)	Chronic	VAS < 2	—	—	Forward bending and backward return	Kinematic variability Local stability Orbital Stability	MeanSD, CV and VR Lyapunov Exponents, Floquet multipliers	Decreased SMV in the long term in LBP. No difference in the short term
Increased SMV									
Williams (2013)	Cross-sectional (Acute LBP vs Chronic LBP)	Chronic	VAS: Acute LBP: 6.2 ± 1.9	TSK: Acute LBP: 39.3 ± 4.1	Mechanical	Forward and backward return, side-bending, twisting, and object lifting	Movement irregularity	Angular velocity-ROM plots	Increased SMV during most tasks in chronic LBP compared to acute LBP
			Chronic LBP: 4.6 ±2.2	Chronic LBP: 38.3 ±7.5					
Ippersiel (2018)	Cross sectional (LBP vs Healthy)	Sub-acute/chronic	NRS: 3.4 (1.1)	—	STarT Back: 4.4 (1.8)	STS	Coordinative variability	CRP curve	Increased SMV over the full STS movement. Increased SMV was more prominent in the start period of STS (the period between onset of STS and the point of loss of contact with the seat) in LBP
			ODI: 25.3 (7.4)						
Silfies (2009)	Cross-sectional (LBP vs Healthy)	LBP	NRS: 3.8 (2.2)	—	Mechanical	Forward bending and backward return	Coordinative variability	CRP curve	Increased SMV during the task in LBP. Greater increase of SMV during backward return than during forward bending in LBP
			RMQ: 8.1 (5.2)						
Bauer (2015)	Cross-sectional (LBP vs Healthy)	Sub-acute/chronic	NRS: 3.4 (1.5)	—	STarT Back < 4	Object lifting	Predictability and/or Complexity	Recurrence rate, Determinism	Increased SMV with increasing LBP intensity
			ODI > 8						
No differences in SMV									
Chehrehrazi (2017)	Cross-sectional (LBP vs Healthy)	Chronic	VAS < 2	—	—	Forward bending and backward return	Goal equivalent variability	Goal equivalent manifold	No differences in SMV between groups
Pranata (2018)	Cross-sectional (LBP vs Healthy)	Chronic	LBP with low disability	—	—	Object lifting	Coordinative variability	CRP curve	No differences in SMV between groups
			NRS: 3.0 ± 1.6						
			ODI: 13.2 ± 4.9						
			LBP with moderate-high disability						
			NRS: 4.5 ± 1.9						
			ODI: 34.4 ± 10.9						
Asgari (2017)	Cross-sectional (LBP vs Healthy)	Chronic	—	—	—	Object lifting	Local Stability	Lyapunov Exponents	No differences in SMV between groups
Graham (2014)	Cross-sectional (LBP vs Healthy)	Sub-acute/chronic	RMQ: 4.0 (2.1)	—	—	Forward bending and backward return	Local Stability	Lyapunov Exponents	No differences in SMV between groups
			ODI: 7.8 (3.5)						
Moreno (2018)	Cross-sectional (LBP vs Healthy)	Chronic	VAS: LBP with non-athletes: 3.9 (1.7)	—	—	Object lifting	Local Stability	Lyapunov Exponents	No differences in SMV between groups
			LBP with athletes: 4.5 (1.8)						

LBP: low back pain; NRS; numerical rating scale; ODI: Oswestry Disability Index; ROM: range of motion; VAS: visual analog scale; RMQ: Roland Morris Disability Questionnaire; TSK: Tampa scale of kinesiophobia; PCS: Pain Catastrophizing Scale; STAI: State-Trait Anxiety Inventory; STS; sit-to-stand-to-sit: CRP: continuous relative phase; ROM: range of motion; SD: standard deviation; CV: coefficient of variation; VR: variance ratio.

Table 4 presents a summary of prospective studies that assessed the changes in SMV in people with LBP [21–23, 55]. Two studies [21, 22] reported increased SMV in some patients and decreased SMV in others, with the changes being associated with reduction of LBP following oral analgesia [21] or neuromuscular exercise [22]. In another prospective cohort study [55] and an interventional study involving random-perturbation therapy [23], no change in SMV was reported despite successful reduction in LBP.

**Table 4:** Summary of prospective studies that assessed the changes in SMVs of LBP patients.

Study	Study Design	LBP characteristics				Task performance	Types of SMV	Statistical tool	Results
		Chronicity	Pain/disability level	Psychological factor	Others				
Significant changes in SMV									
Williams (2014)	Intervention study	Acute and Chronic	VAS: Acute 6.2 ± 1.7	TSK: Acute 39.0 ± 4.8	Mechanical	Forward and backward return, side-bending, twisting, and object lifting	Movement irregularity	Angular velocity-ROM plots	Oral analgesia decreased SMV during forward bending in the acute LBP group, and increased SMV during side bending in the chronic LBP group. % changes (from pre- to post-intervention): N/A
	Oral analgesia: self-administered between pre and post-movement trials		Chronic 4.6 ± 2.2	Chronic 38.9 ± 6.9					
Bauer (2019)	Intervention study	Sub-acute	VAS: At baseline	—	—	Object lifting	Predictability	Determinism	The SMV showed a treatment effect after the 6-month NME intervention.
	NME group: training focused on balance, coordination, endurance, and strength of trunk, aiming to increase lumbar movement patterns available. Sessions of 60 min twice a week for 6 months		NEM group: 3.4 (2.1)						% changes (from pre- to post-intervention):
									Predictability of AD:
			Control group: 2.8 (2.1)						NME group: −1.8%
									Control group: 5.0%
			At post-intervention						Predictability of AV:
									NME group: 0.1%
	Control group: no intervention		NME group: 2.5 (2.0)						Control group: 7.8%
			Control group: 2.8 (1.9)						
No changes in SMV									
Shojaei (2019)	Prospective cohort study	Acute/subacute (Non-chronic)	VAS Moderate-severe LBP ≧ 4	—	—	Forward bending and backward return	Coordinative variability	CRP curve	SMV in both LBP groups tended to be lower than those of the control group
			Low-moderate LBP < 4						The lower SMV in both LBP groups was sustained over time despite significant improvements in LBP intensity and disability
Adamantios (2017)	Intervention study	Chronic	VAS	—	—	Object lifting	Local stability	Lyapunov Exponents	No significant changes in SMV following interventions despite a reduction in LBP
	A random-perturbation therapy group: device that induced disturbances in the anteroposterior and mediolateral axes of the trunk. 26 sessions of 1.5 h twice a week for 13 weeks.		At baseline						% changes (from pre- to post-intervention)
			Perturbation-based group: 4.0 (1.4)						
			Control group: 4.2 (1.7)						A random-perturbation therapy group: 3.5%
			At post-intervention						
			Perturbation-based group: 3.0 (1.9)						Control group: −6.8%
	Control group: no intervention		Control group: 3.9 (1.9)						

AD: angular displacement; AV: angular velocity; LBP: low back pain; NRS; numerical rating scale; ODI: Oswestry Disability Index; ROM: range of motion; RQA: recurrence quantification analysis; VAS: visual analog scale; RMQ: Roland Morris Disability Questionnaire; TSK: Tampa scale of kinesiophobia; NME: neuromuscular exercises; RPT: random-perturbation therapy.

## Discussion

### Methodologies used to assess SMV

This study found that the most commonly used linear statistical tool was the CRP curve for calculating coordinative variability [8, 18, 50, 52, 53]. Coordinative variability reflects the degree to which two segments of the body (e.g., spine and pelvis) can move in the same or opposite direction [10]. Coordinative variability has been used as an outcome measure to investigate risk factors for overuse injuries in other parts of the body [10], including the upper limbs [58], knees [59, 60], and feet [61]. A recent systematic review [62] found that 60% of studies assessing coordinative variability identified a significant difference between individuals with and without lower limb injuries [62]. Notably, all studies [8, 18, 50, 53], except one [52], reported a significant difference in coordinative variability between individuals with and without LBP. Furthermore, as shown in S2 Table, studies using the CRP curve presented moderate-to-excellent reliability [8, 18, 52]. These indicate that the CRP curve can be a promising statistical tool in comparing the SMV of individuals with and without LBP.

In terms of non-linear statistical tools, the Lyapunov exponent analysis [16, 23, 48, 49, 51] was commonly used. The Lyapunov exponent is considered as a measure of local dynamic spine stability and associates with mechanical spine stiffness, as shown by the EMG-driven biomechanical spine model [7]. Evidence found that local dynamic spinal stability was perturbed during repeated trunk movements during conditions of fatigue [63], while lifting heavy loads [7], and at high speeds [51] in healthy individuals. Of note, these conditions commonly occur in daily activities, workplace settings, and sports, and are often associated with LBP [64]. In this review, four studies [6, 16, 19, 56] using non-linear statistical tools found significant differences in SMV between individuals with and without LBP during repeated lifting [19, 56] or forward bending and backward return tasks [6, 16]. Because non-linear tools describe a series of measurements taken at specific intervals over uninterrupted time, they may be suitable for assessing the functional adaptability of the trunk in the presence of LBP through the performance of LBP-relevant cyclic movements [5, 15].

There is limited information as to whether one tool is superior to the others because different concepts of variability and analysis techniques exist within and between the tools. For example, one study [16] used both linear and non-linear statistical tools to compare individuals with and without LBP, and found no difference in the amount of variability, but a significant difference in the structure of variability. It may be possible that non-linear statistical tools may have advantages in quantifying the adaptability of neuromotor behavior in individuals with LBP [47]. However, this review identified a higher percentage of studies that found significant differences in SMV between patients with and without LBP using linear rather than non-linear statistical tools (75% vs. 57%, respectively). This may suggest that linear tools provide higher sensitivity in comparing the SMVs of individuals with and without LBP. Future research should include a series of diagnostic studies, including the accuracy and effectiveness of both tools that measure SMV in LBP patients [65].

### SMV with and without LBP

Several studies reported differences in SMV both between individuals with and without LBP, and within the LBP population [6, 8, 16, 18, 19, 50, 53, 54, 56]. Changes in SMV with the reduction of LBP were also found [21, 22]. These findings support the assumption that SMV may be associated with LBP [5]. However, the direction of change was inconsistent among the studies, indicating the importance of considering the heterogeneity of LBP, and dividing LBP patients into subgroups based on LBP type.

The first potential subgroup would include LBP individuals with decreased SMV compared with those without LBP [6, 8, 16, 19, 53, 54]. The common features of this subgroup include acute or sub-acute LBP [6, 53, 54], mild-to-moderate fear of movement [19], and moderate LBP intensity [54]. These characteristics may be a consequence of increased co-contraction of trunk muscles as an adaptive strategy to avoid nociceptive excitation, pain, or injury, or as an anticipation of such threats to prevent further injuries [20, 66]. However, the decreased SMV may also cause unnecessary spinal compressive loads and muscle fatigue [67, 68]. Interestingly, a prospective study [55] found that the decreased SMV in non-chronic LBP patients persisted over 6 months even after remission of LBP. This may indicate that decreased SMV is unlikely to improve during the natural course of LBP and may thus contribute to the recurrence of LBP if left untreated. This speculation needs to be clarified in future studies.

The second potential subgroup would consist of LBP patients with increased SMV compared with those without LBP, with common features being chronic LBP [50, 54, 56] or mechanical LBP [18, 54]. Such increased SMV may reflect poor movement control within the neutral zone, and the increase in tissue strain associated with loading during end-range postures and movements [11, 12]. However, increased variability has also been observed in healthy subjects during fatigue-inducing tasks such as repetitive lifting [69], throwing [70, 71], and reaching [72]. Therefore, an increase in SMV may also reflect a positive strategy of exploring spinal movement solutions to minimize fatigue and injuries and preserve task performance [4, 10].

The third potential subgroup would involve LBP subjects without SMV differences as compared to those without LBP [17, 48, 49, 51, 52]. Features of this subgroup include sub-acute or chronic LBP [17, 48, 49, 51, 52], relatively low pain intensity [17], and a low level of disability [49]. Furthermore, it may be hypothesized that this subgroup associates with dominant social and psychological characteristics that contribute to LBP, and is thus independent of changes in SMV [3]. However, given that studies reporting such findings [17, 48, 49, 51, 52] did not include measures of psychosocial status, such hypotheses cannot be discussed further, and future investigations are required.

Aside from SMV differences according to LBP type, the current review found considerable differences with respect to task conditions. It is conceivable that different tasks that require different mechanical loads may influence SMV [73]. The included studies found decreased SMV during asymmetric [8], high-velocity [8], long-term repeated trunk movement [16], and during fatigue-inducing protocols [6]. Interestingly, two studies [8, 16] only included LBP subjects with pain intensities lower than 2/10 on the visual analogue scale. These results indicate that biomechanically demanding tasks may induce protective mechanisms that decrease SMV even if the LBP levels were relatively low. In contrast, another study [50] found an increase in SMV during the starting period of a sit-to-stand-to-sit (STS) test (the period between STS onset and the point of loss of contact with the seat) compared to other periods in LBP subjects with low-to-moderate disability (average Oswestry Disability Index score, 25.3%). It has been shown that the starting period of the STS test requires lower mechanical demands with less trunk and lower limb muscle activities compared to other periods of the STS [74]. Thus, it is possible that when a movement task is not mechanically demanding, increased SMV due to impaired sensory feedback and/or decreased motor commands become more prominent over the protective behaviors [75]. Therefore, it is plausible that the same LBP population could show an increase or decrease in SMV depending on task demands.

Lastly, this review highlighted that the interaction between tasks and LBP characteristics may represent important contributing factors of SMV in LBP patients. For example, a study [8] reported decreased SMV among LBP subjects only in the combination of asymmetric and high-velocity conditions. Therefore, the following can be assumed: (1) the combination of higher acute LBP, fear of movement, and high demand tasks (high speeds, high repetitive movements, and fatigue-inducing conditions) could increase the possibility of decreased SMV; (2) the combination of chronic LBP and mechanical pain states, and lower mechanical demands could increase the possibility of increased SMV; and (3) the lack of these combinations may not result in any SMV changes. Further research on these interactions is required.

### Methodological concerns

Methodological heterogeneity and limitations were found among the studies included in this review. The review found a high risk of selection bias with regards to the LBP groups, resulting from problems such as (1) lack of reporting of disability levels [6, 8, 16, 17, 21–23, 48, 51, 53–56], (2) relatively low pain intensity among the participants [8, 16, 17, 49, 51], (3) lack of a set of procedures for the diagnosis of LBP [6, 17, 19, 22, 50, 51, 53, 56], and (4) multiple or unclear sample sources [6, 19, 23, 50, 51, 53, 56]. Similarly, only a few studies [16, 19, 52, 53] showed evidence that the healthy controls used had never experienced LBP. It is possible that some controls had a history of LBP, potentially altering their motor control strategies [76]. Such an issue could result in the LBP and control groups being inadequately representative, rendering it inappropriate to extrapolate the results to other patient populations.

### Strategies to modify the SMV

As shown in Table 4, this review found three interventions that aimed to optimize SMV: oral analgesia [21], neuromuscular exercises [22], and random-perturbation therapy [23]. Of these, only one RCT applied the 6-month neuromuscular exercise and the wait-and-see intervention arms on subacute LBP patients and demonstrated SMV differences immediately after the intervention period between the two arms [22]. The neuromuscular exercises were specifically designed to increase SMV by requiring stability and mobility of the spine while executing a variety of trunk and lower limb movements [22, 77]. Thus, neuromuscular exercises could be a possible intervention to change and optimize SMV in certain subgroups of LBP patients. Further investigations on this are required.

### Clinical implications

A clear insight into the nature and mechanisms underlying the alterations of motor control strategies may lead to effective diagnosis and interventions in LBP patients. This review suggested the importance of SMV in LBP patients for these purposes. Of note, several studies [6, 21, 22, 53–56] used wearable movement sensors to assess SMV. These sensors may allow clinicians and researchers to easily and accurately assess SMV, which may increase the applicability of assessing SMV in clinical settings.

However, this review also suggested the consideration of multiple contributing factors to the alteration of SMV in LBP patients: (1) the selection of statistical tools; (2) LBP characteristics including chronicity, mechanical or no mechanical states, LBP intensity and psychological characteristics; and (3) biomechanical movement task demands. Although potential subgroups according to the direction of change in SMV and factors that contributed to these subgroups have been proposed, interpretation regarding whether the alterations of SMV are positive or negative strategies for LBP remain unknown, as the included studies were largely cross-sectional design [6, 8, 16–19, 48–54, 56]. Future research is required to reliably identify the subgroups that respond consistently and favorably to targeted interventions to optimize SMV in LBP patients [24].

### Limitations

A limitation of this scoping review is that the included studies were limited to peer-reviewed papers in English, introducing the possibility of publication bias and the exclusion of other relevant data. Nevertheless, we believe that the suggestions for future research derived from the current review would not change if other studies were included, because a large number of published studies were screened and considered for inclusion. Furthermore, this review only included studies that investigated SMV in LBP population using motion capture devices. Since makers or sensors were placed on the skin, it might be possible that SMV had been affected by errors due to soft tissue artifacts, which may conceal differences between groups [78].

## Conclusions and suggestions for future research

The existing literature has provided useful indications for future research. First, this review identified two types of statistical tools used to assess SMV, with each type evaluating functionally different aspects of SMV. This should therefore be considered in the study design stage of future research. Second, this review proposed three potential LBP subgroups with different SMV characteristics, with each subgroup reflecting different motor strategies depending on LBP characteristics and task demands. Since the identification of differences in motor control strategies is important for effective diagnosis and personalized treatment [2], assessing the association of SMV with clinical variables such as pain/disability levels and psychological characteristics is necessary. Third, there is preliminary evidence from one RCT that neuromuscular exercises could modify SMV. Thus, the current review suggests a rationale for well-designed RCTs involving neuromuscular exercise interventions.

## Supporting information

S1 File Search strategy on Medline. (TIFF)

S2 File Modified version of the NOS. (TIFF)

S1 Table Excluded studies. (TIFF)

S2 Table Summary of key methodology. (PDF)
