# Supplementary figures and images for: Spinal movement variability associated with low back pain: A scoping review
Source: PLoS One. 2021 May 24;16(5):e0252141. doi: 10.1371/journal.pone.0252141 (PMC8143405; doi:10.1371/journal.pone.0252141)

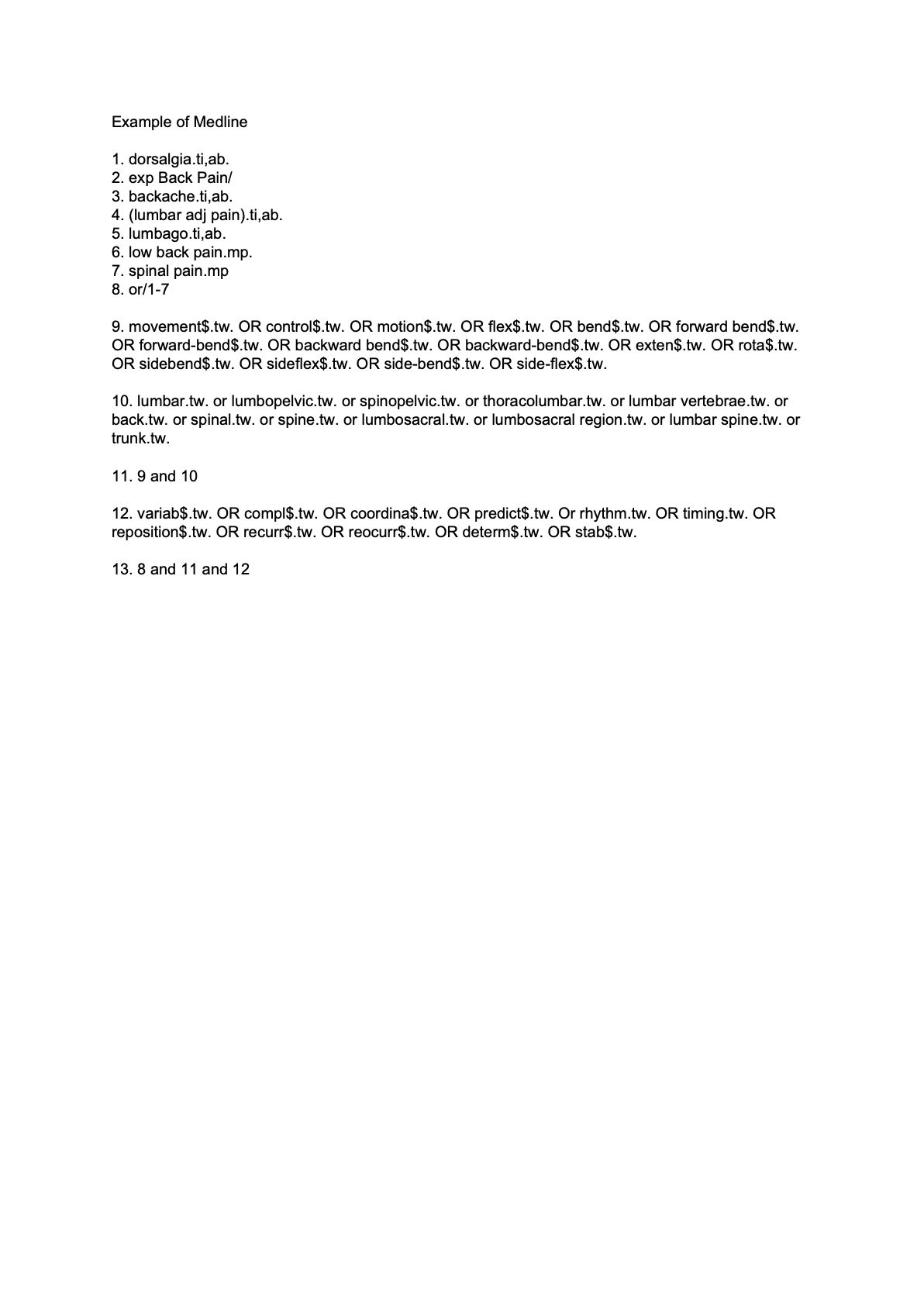

Supplement: S1 File — (TIFF) [file pone.0252141.s001.tiff]

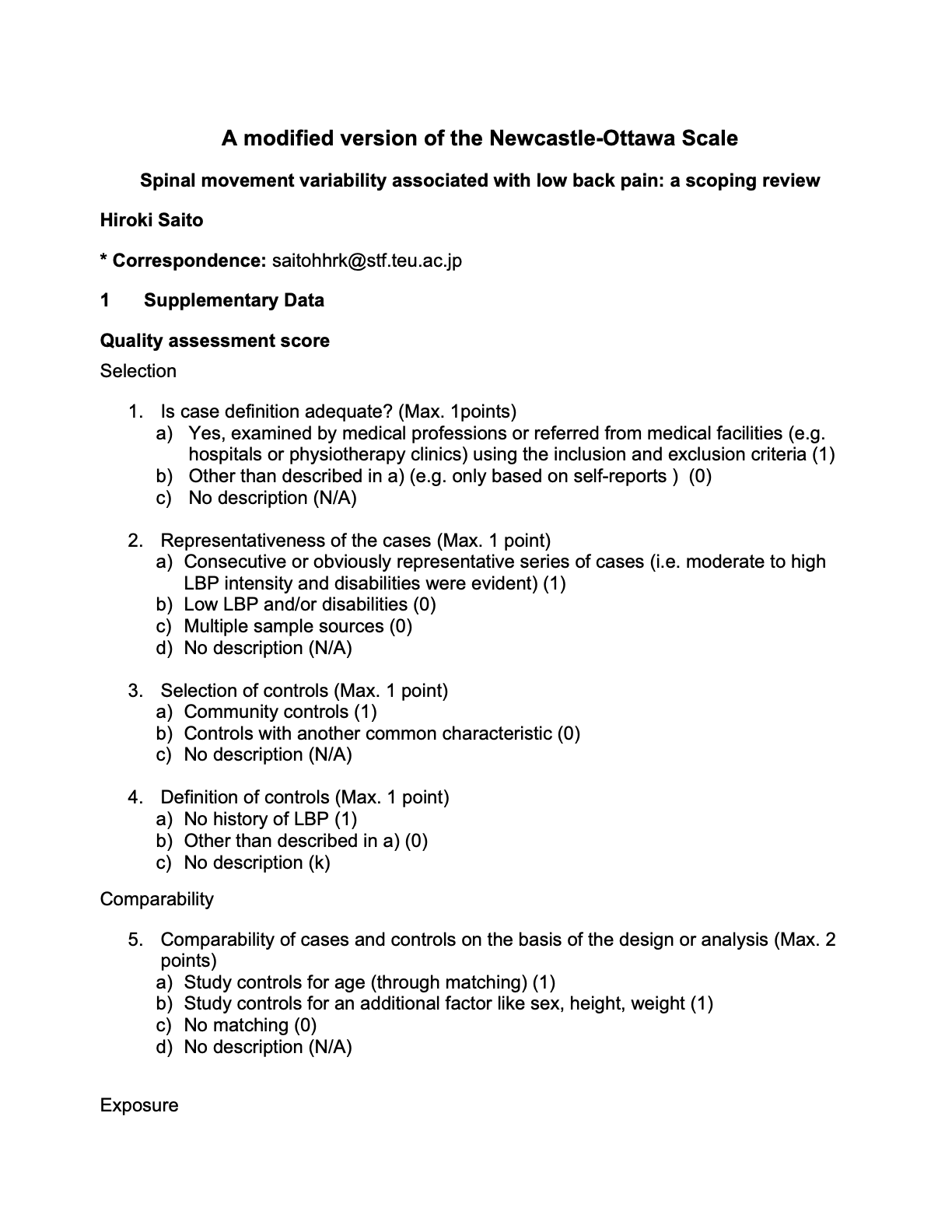

Supplement: S2 File — (TIFF) [file pone.0252141.s002.tiff]
