# Supplementary material for: Spinal movement variability associated with low back pain: A scoping review
Source: PLoS One. 2021 May 24;16(5):e0252141. doi: 10.1371/journal.pone.0252141 (PMC8143405; doi:10.1371/journal.pone.0252141)
Supplement: S2 Table — (PDF) [file pone.0252141.s004.pdf]

					Population						Task perfomance			Measument			Analysis characteristics			Results	Others		
ID	Authors	year	Titiles	Study type	Participants(n,age,gender)	Sample source	Eligibility criteria	Symptom duration	Classification (Duration, tyep)	Pain/dasability level/Psychological feature	Task perfomance	Condition	Number of repetition,time	Measurement Apparatus	Sensor/maker placements	Angle definition	Analysis technique	Analysis metric	Type of variability	Main results	Founding source	Reliable data acquisition	Power calculation
172	Adamantios	2017	A random-perturbation therapy in chronic non-specific low-back pain patients: a randomised controlled trial	Intervention<br><br>A random-perturbation therapy : Device that induced disturbances in the anteroposterior and mediolateral axes of the trunk. 26 sessions of 1.5 h twice a week for 13 weeks.<br><br>Sessions of 60 min twice a week for six months.	n = 45<br>(Chronic LBP who received perturbation-based intervention = 24, Chronic LBP who received no intervention = 21)<br><br>Age and gender :<br>LBP who received NME : 0/42 (m/f), 45.7 (±7.8) years<br>LBP who received no intervention : 0/41 (m/f), 46.7 (±7.7) years	Unclear	Inclusion for LBP<br>・Inclusion criteria were: 18–50 years; low-back pain at least since 12 weeks before the recruitment and at least on half of the days; no recognizable, specific pathology based on infection, structural deformity, tumor, trauma or inflammatory disorder; moderate low-back pain (VAS 2–6); and restriction during daily activities.<br>・Non-specific chronic low-back pain following the recommendation of the "German National Disease Management Guideline for Low-back pain" of the German National Program for Disease Management Guidelines	> 3 months	Chronic	Pain intesity(VAS)<br>At baseline<br>Perturbation-based group : 3.96 (1.41)<br>Control group : 4.22 (1.66)<br><br>At post-intervention<br>Perturbation-based group : 2.98 (1.85)<br>Control group : 3.93 (1.91)	Lifting an object	Execute repetitively a lifting task between two tables (53 and 90 cm height) with a fre- quency of 12 cycles per minute (0.2 Hz) holding a pot with a mass of 1.5 kg. The patients performed the repetitive lifting task starting from the 90 cm table, placing the pot on the 53 cm table, and then placing it back.	40 cycles	Vicon, Oxford, UK	Iliac spines anterior superior left and right and iliac spines posterior superior left and right) and of a marker triad (radius 6 mm) located at the 12th thoracic vertebra (T12)	Thorax-lumber	Lyapunov analyses	LE	Local stability	There was neither a main effect of time (p = 0.468) nor a time by group interaction (p = 0.257) for the maximum Lyapunov exponent (λmax). After the 13 weeks, the pain score decreased significantly in the perturbation-based intervention group (p = 0.002), while the control group did not show any differences.	The German Federal Institute of Sport Science on behalf of the Federal Ministry of the Interior of Germany as the major funder.	Not clear	Yes
69	Asgari	2015	The effects of movement speed on kinematic variability and dynamic stability of the trunk in healthy individuals and low back pain patients	Cross - sectional	n = 26<br>(Chronic LBP = 14 Contol = 12)<br><br>Gender and age<br>LBP : 14/0 (m/f), 31.5 (±6.6) years<br>Control : 12/0 (m/f), 28.0 (±4.4) years	LBP:<br>Local physical therapy clinics<br>The physiotherapist confirmed diagnosis<br><br>Control:<br>The University of Social Welfare and Rehabilitation Sciences	Inclusion for LBP<br>・With a pain that is not attributed to any recognizable pathology (e.g. inflammation, tumor, osteoporosis, infection, fracture, etc.) that lasts a duration of at least 3 months<br>・Pain level was lower than two on the 10-point VAS at the time of the test<br><br>Inclusion for Control<br>・No self-reported history of LBP<br><br>Exclusion for both groups<br>・Any background surgical intervention on the vertebral column, specific spinal deformities and any neurological, pulmonary, rheumatism and metabolism diseases	>= 3 months	Chronic	Pain level(VAS) < 2	Forward bending and backward return	They were asked to touch a target with their hands located in the sagittal midline, 50 cm anterior to the knee in upright position, while looking at another target placed at shoulder height in the sagittal midline during standing.<br>Specifically, the participants were asked to touch the lower target with extended arms, followed by looking at the upper target with enough extended trunk while their arms were positioned alongside their bodies.<br><br>Three conditions:<br>・20 cycles/min<br>・self-selected<br>・40 cycles/min	30 cycle	Vicon, Oxford, UK	Vicon plug-in gait instructions	Trunk	Kinematic variability<br>Lyapunov analyses<br>Floquet multiplier(FM) method	the MeanSD, CV and VR of instaneous angular displacement<br>Short term LE<br>Long term LE	The amout of variability<br>Local stability	No difference in Mean SD, CV, and VR<br>No difference in the short term LE<br>Significant main effect of group on showed the long term LE in LBP was lower than control (F = 5.01 p = 0.03)<br>No group effect on FM	A research grant from University of Social Welfare and Rehabilitation Sciences, Tehran, Iran. The authors declare that there is no conflict of interest that could inappropriately influence this work	Not clear	No
131	Asgari	2017	Local dynamic stability of the spine and its coordinated lower joints during repetitive Lifting: Effects of fatigue and chronic low back pain.	Cross - sectional	n = 28<br>(Chronic LBP = 14 and Conrol 14)<br><br>Age and gender :<br>LBP : 32/31 (m/f), 39.5 (±7.0) years<br>Control : 14/17 (m/f), 40.1 (±12.1) years	LBP<br>Rehabilitation and physiotherapy centers<br><br>Control<br>The students and staff of universities	Inclusion for LBP<br>・20 or more years of age<br>・Had a history of pain and discomfort in the lumbar area without pain referring to the lower limbs<br>・Experienced CLBP recurrently for more than three months with no recognized pathology<br><br>Exclusion for LBP<br>・Symptoms for nerve root irritation, vertebral or leg fracture and/or history of surgery, neurological or rheumatic disease and an observable spinal deformity<br><br>Inclusion for Control<br>No self-reported history of CLBP	> 3 months	Chronic	N/A	Lifting a dumbell	Lift two equally-sized dumbbells having a combined weight equivalent to 15% of their body weight. The dumbbells were lifted in the hands up waist level while standing upright. The participants were asked to lower the dumbbells to the floor and lift and lower the loads repeatedly. Participants were allowed to perform the repetitive lifting at their preferred speed while moving their lower limbs freely. The initial 20 cycles and the last 20 cycles of each angular time series were considered to be the early- and late-fatigue conditions.	Until participant reported a score of 17 on the Borg scale	Vicon; Oxford Metrics; UK	the standard Vicon plug-in-gait on the lateral malleolus, me- tatarsal bone, calcaneus, lateral femoral condyle, anterior superior iliac spine, posterior superior iliac spine, T10 and C7 spinous processes and xiphoid process.	Thorax-pelvis	Lyapunov analyses	LE	Local stability	No difference in LE between groups<br>No interaction between LBP and fatigue on LE	N/A	Not clear	No
125	Bauer	2015	Pain intensity attenuates movement control of the lumbar spine in low back pain	Cross - sectional	n = 94<br>(Sub-acute/Chronic LBP = 63 contol = 31)<br><br>Age and gender :<br>LBP : 32/31 (m/f), 39.5 (±7.0) years<br>Control : 14/17 (m/f), 40.1 (±12.1) years	LBP:<br>Recruited from physiotherapy practice, the university campus and through newspaper advertisements<br>Unclear regading who conformed diagnosis<br><br>Control:<br>Recruited from physiotherapy practice, the university campus and through newspaper advertisements	Inclusion for LBP<br>・Current episode of LBP persisted for four weeks or longer<br>・At least moderate disability, defined as an Oswestry-disability-index (ODI) >8%<br>・A low level of psychosocial risk factors defined with less than four points on the subscale of the STarT Back screening tool<br><br>Inclusion for Control<br>・No current LBP episodes or episodes during the preceding three months<br><br>Exclusion for both group<br>・Specific LBP<br>・Vertigo or disturbance of the equilibrium<br>・Systemic diseases (diabetes, tumours)<br>・Pain in other areas of the body (neck, head, thoracic spine, or arms)<br>・Complaints, injury, or surgery of the legs (hips to feet) within the last six months<br>・Medication affecting postural control (e.g. anti-depressants)<br>・Pregnancy	≧ 4weeks	Sub-acute and chronic	ODI > 8<br>Pain level (NRS) : 3.4 (1.5)<br>STarT Back < 4	Lifting a box	The box was loaded to ten percent of their body weight. Four seconds duration, starting in upright standing. During each cycle the participants were asked to pick up the box from the ground and put it back down again.	10 cycle	IMU sensor (Valedo®Motion, Hocoma AG, Volketswil, Switzerland)	The sacrum(S2) and L1	Lumbar – pelvis	Recurrence quantification analysis	REC,DET	Predictability and/or Complexity	DETAD significantly decreased with increasing LBP intensity (p = 0.01)<br>RECAD was not significantlt affected by LBP intensity<br><br>DETAV significantly decreased with increasing LBP intensity (p = 0.03)<br>RECAV significantly decreased with increasing LBP intensity (p = 0.05)<br><br>DETAD significantly decreased with increasing LBP intensity (p = 0.05)<br>RECAA either increased or decreased with increasing LBP intensity, depending on the age of the participant	The Swiss Commission for Technological Innovation (project 12413.1). Dr Rissanen was supported by the Academy of Finland (project 252748). The sponsors had no influence on the conduct of the study	Not clear in this study. However, another study (bauer 2015) found the high reliability.<br><br>Bauer CM, Rast FM, Ernst MJ, Kool J, Oetiker S, Rissanen SM, et al. Concurrent validity and reliability of a novel wireless inertial measurement system to assess trunk movement. J Electromyogr Kinesiol. 2015;25: 782–790.	No
66	Bauer	2017	The effect of muscle fatigue and low back pain on lumbar movement variability and complexity	Cross - sectional	n = 86<br>(Sub-acute/Chronic LBP = 59 contol = 27)<br><br>Gender and age<br>LBP : 30/29 (m/f), 39.1 (±12.8) years<br>Control : 12/15 (m/f), 39.6(±11.6) years	LBP:<br>Physiotherapy practices, the university campus and through newspaper advertisements<br>Unclear regading who conformed diagnosis<br><br>Control:<br>Physiotherapy practices, the university campus and through newspaper advertisements	Inclusion for LBP :<br>・a current episode of sub-acute or chronic non-specific LBP that persisted for four weeks or longer;<br>・a mean LBP intensity of !1 on NRS over the last four weeks;<br>・a moderate disability defined as an Oswestry-disability-index (ODI) > 8%;<br>・a low level of psychosocial risk factors defined with less than four points on the psychosocial subscale of the STarT Back screening tool<br><br>Inclusion for Control<br>・Without history of a LBP episode during the preceding three months<br><br>Exclusion for both groups<br>・Specific LBP<br>・Vertigo or disturbance of the equilibrium<br>・Systemic diseases (diabetes, tumours)<br>・Pain in other areas of the body (neck, head, thoracic spine, or arms)<br>・Complaints, injury, or surgery of the legs (hips to feet) within the last six months<br>・Medication affecting postural control (e.g. anti-depressants)<br>・Pregnancy	≧ 4weeks	Sub-acute and chronic	ODI > 8<br>Pain level (NRS) ≥ 1<br>STarT Back < 4	Flexion and Extension (sitting)	During each cycle the participants were asked to flex and extend their lumbar spine and hip as far as possible whilst adhering to the timed window.Tests were perfomed pre and post fatiguing of the lumbar musculature<br><br>Two conditons:<br>・Pre/post fatigue	60sec	IMU sensor (Valedo®Motion, Hocoma AG, Volketswil, Switzerland)	The sacrum(S2) and L1	Lumbar – pelvis	Recurrence quantification analysis	DET,SaEn	Predictability and/or Complexity	Significant group-fatigue effect (95% HPDI) revealed %DETAV decreased and SaEnAV increased in control following fatigue protocol while in LBP they did not change. No significant group-fatigue effect of %DETAD and SaEnAD<br><br>%DETAD : - 0.6 (- 2.4 to 1.0)<br>SaEnAD : - 0.1 (- 0.4 to 0.2)<br>%DETAV : 3.7 (2.3 to 7.1)<br>SaEnAV : - 1.4 (- 2.7 to - 0.1)	The Swiss Commission for Technological Innovation (project 12413.1). The sponsors had no influence on the conduct of the study	Not clear in this study. However, another study (bauer 2015) found the low reliability.<br><br>Bauer CM, Rast FM, Ernst MJ, Kool J, Oetiker S, Rissanen SM, et al. Concurrent validity and reliability of a novel wireless inertial measurement system to assess trunk movement. J Electromyogr Kinesiol. 2015;25: 782–790.	No
139	Bauer	2019	Efficacy of six months neuromuscular exercise on lumbar movement variability - A randomized controlled trial	Intervention<br><br>NME : Trainings focused on balance, coordination, endurance and strength of trunk. Sessions of 60 min twice a week for six months.	n = 83<br>(Sub-acute LBP who received Neuromuscular exercise = 42, Sub-acute LBP who received no intervention = 41)<br><br>Age and gender :<br>LBP who received NME : 0/42 (m/f), 45.7 (±7.8) years<br>LBP who received no intervention : 0/41 (m/f), 46.7 (±7.7) years	LBP groups :<br>University Hospital	Inclusion for LBP<br>・Age between 30 and 55 years<br>・Working at current job for at least 12 months<br>・Suffering from LBP during the past four weeks, with a mean minimum intensity of two points on NRS<br><br>Exclusion for LBP<br>・Serious former back injury (fracture, back surgeries, disc protrusions)<br>・Chronic LBP defined by a physician<br>・Self-reported continuous LBP of seven months or longer duration<br>・Other diseases or symptoms that limit participation in moderate intensity NME<br>・Current engagement in NME more than once a week<br>・Pregnant or postpartum within the past twelve months	< 4 weeks	Sub-acute	Pain intesity (VAS)<br>At baseline<br>NEM group : 3.40 (2.10)<br>Control group : 2.80 (2.11)<br><br>At six month<br>NEM group : 2.52 (1.99)<br>Control group : 2.75 (1.90)<br><br>At 12 months<br>NEM group : 2.13 (2.00)<br>Control group : 2.44 (2.27)	Lifting a box	The participant was required to squat, pick up a box from the ground and then return to the squat position, whilst guided by a metronome set at 50 bpm.The box weight was set at 10% of the participant's body weight.	5	IMU sensor (ValedoMotion, Hocoma AG, Volketswil, Switzerland)	The sacrum(S2) and L1	Lumbar – pelvis	Recurrence quantification analysis	DET	Predictability and/or Complexity	Lumbar movement variability showed a treatment effect after the six months of NME intervention. In the NME group, DET AD decreased and DET AV remained constant throughout the intervention phase. Both DET AD and DET AV increased in the control group between the three points in time<br><br>Baseline:<br>DETAD NME:65.5±11.1<br>DETAD control:63.7±9.7<br><br>DETAV NME:64.8±11.4<br>DETAV control:63.7±9.1<br><br>At six month:<br>DETAD NME:64.3±12.1<br>DETAD control:66.9±9.6<br><br>DETAV NME:64.9±12.1<br>DETAV control:68.7±6.5<br><br>At twelve months:<br>DETAD NME:66.7±13.8<br>DETAD control:68.8±7.0<br><br>DETAV NME:66.7±13.8<br>DETAV control:68.8±8.0	The Social Insurance Institution of Finland 37/26/2011 and Pirkanmaa Hospital District, Tampere, Finland 9K127 and 9M099	Not clear	No
65	Chehrehrazi	2017	Goal equivalent manifold analysis of task performance in non-specific LBP and healthy subjects during repetitive trunk movement: Effect of load, velocity, symmetry	Cross - sectional	n = 44<br>(Chronic LBP = 22 contol = 22)<br><br>Gender and age<br>LBP : 19/11 (m/f), 23.9 (±3.3) years<br>Control : 13/12 (m/f) , 27.4 (±5.1) years	LBP:<br>The university community<br>Unclear regading who conformed diagnosis<br><br>Control:<br>The university community	Inclusion for LBP<br>・Episodic LBP for 12 months or more with pain level lower than two on the 10-point VAS at the time of testing<br><br>Inclusion for Control<br>・No history of LBP limiting their function within the previous year<br><br>Exclusion for both groups<br>If they had (1) structural deformities of the spine, (2) pain extending to and/or beyond the gluteal fold, (3) previous spine, hip or knee surgery, (4) spinal root pain, (5) spinal tumors or infections (6) neurologic symptoms such as weakness, tingling or anesthesia in lower extremities, and (7) visual or hearing impairment	≧ 12 months	Chronic	Pain level(VAS) < 2	Forward bending and backward return	A flexion limit adjusted so that subjects needed to touch it at their knee height with their hands. Another limit was a sign located on the wall at eyes' level that subjects were asked to see it when they returned to the upright position. Movement was synchronous with a metronome.<br><br>Six conditiions<br>- Symmetric/asymetric (with right rotation)<br>- Velocity high (40cyles per min)/low (20cyles per min)<br>- No-load/loaded (8kg)	30	Vicon, Oxford, UK	Vicon plug-in gait instructions.	Trunk	GEM	SD of Variability along GEM(δt)<br>SD of Variability perpendicular GEM (δp)<br>Ratio SD (δt)/SD (δp)	Goal equivalent variability	No main effec of group<br>No interaction effect : Group(LBP and control), Symmetry(symmetric vs. asymmetric), Velocity(high vs. low), load (no-load vs. loaded)	Partial funding and support were provided by Tehran University of Medical Sciences (TUMS) as a PhD grant for one of the authors	Not clear	No
111	Dideriksen	2014	Deterministic accessory spinal movement in functional tasks characterizes individuals with low back pain	Cross - sectional	n = 34<br>(Chronic LBP = 17 contol = 17)<br><br>Gender and age<br>LBP : 41%/59% (m/f), 32.5 (±9.6) years<br>Control : 47%/53% (m/f), 29.7 (±7.3) years	LBP:<br>A Pain Clinic, general practitioners or through general advertising in the popular press<br>Unclear regading who conformed diagnosis<br><br>Control:<br>Not clear	Inclusion for LBP<br>・Suffering from non-specific episodic LBP lasting longer than three months with periods of symptom aggravation and remission in the last six months<br>・Lasted at least one week with sufficient intensity to limit function<br><br>Inclusion for Control<br>・no relevant history of back or lower limb pain or injury that limited their function and/or required treatment from a health professional)<br><br>Exclusion criteria for both group<br>・Major circulatory, neurological, or respiratory disorders, recent or current pregnancies, previous spinal surgery, current treatment for LBP from health care providers, or participation in specific trunk muscle exercise in the past 3 months<br>・Taking any medication such as opioids, anticonvulsants, or antidepressants<br>・Non-steroidal anti-inflammatory drugs (NSAIDs) on a regular basis	34.2 (±29.3) months<br><br>Inclusion criteria :<br>>3 months	Chronic	ODI : 14.2.(7.2)<br>Pain level(NRS) : 3.1(2.2)<br><br>SF-36 (total) : 66.9 (12.2)<br>Physical : 60.9 (14.2)<br>Mental : 67.6 (14.1)<br>TSK : 31.8 (5.9)<br>PCS : 16.1 (8.5)<br>STAI : 40.2 (7.1)	Lifting a box	Move a box (40 × 20 × 30 cm) with hole-shaped handles, loaded with a weight of 5 kg, between two shelves placed approximately at knee (lateral epicondyle of femur with the knee extended) and shoulder (position of the clavicle while standing) height	3 min	Epionics SPINE, Epionics Medical GmbH, Potsdam, Germany	Each sensor strip measures angles in 12 pre-determined, adjacent, 25-mm long segments fixed lateral to the spine of the subject. Each strip assesses the curvature of the back shape along the 12 connected segments by measuring bending of the segments in the sagittal plane relative to one another using a series of strain gauge sensors. The two sensor strips together are used to quantify movements and rotations outside of the sagittal plane	Segments in the sagittal plane relative to one another(12)	Recurrence quantification analysis	DET	Predictability and/or Complexity	%DET of the acdessory angular trajectories in 8 of 12 sensors was significantly higher in LBP (p < 0.05)<br>%DET of the task related angular trajectories in 1 of 12 sensors was significantly higher in LBP (p < 0.05)	N/A	Not clear	No

					Population						Task perfomance			Measument			Analysis charactaristics			Results	Others		
ID	Authors	year	Titiles	Study type	Participants(n,age,gender)	Sample source	Eligibility criteria	Symptom duration	Classification (Duration, tyep)	Pain/dasability level/Psychological feature	Task perfomance	Condition	Number of repetition,time	Measurement Apparatus	Sensor/maker placements	Angle definition	Analysis technique	Analysis metric	Type of variability	Main results	Founding source	Reliable data acquisition	Power calculation
75	Graham	2014	Comparing the local dynamic stability of trunk movements between varsity athletes with and without non-specific low back pain	Cross - sectional	n = 20<br>(Sub-acute/Chronic LBP = 10 contol = 10)<br><br>Gender and age<br>LBP : 4/6 (m/f), 20.8 (±1.4) years<br>Control : 4/6 (m/f), 20.6 (±1.0) years	LBP:<br>The Queen's University Athletics Department<br>Medical professions confromed diagnosis<br><br>Control:<br>The Queen's University Athletics Department	Inclusion for LBP<br>・Had been experiencing continuous non-specific LBP for at least four weeks and had been treated by a medical professional and/or missed game time due to their back pain<br><br>Inclusion for Control<br>・Had never experienced LBP that lasted for longer than four weeks, had never missed game time due to LBP, and had never had received treatment for LBP<br><br>Exclusion for both groups<br>・Any background surgical intervention on the vertebral column, specific spinal deformities and any neurological, pulmonary, rheumatism and metabolism diseases	N/A<br><br>Inclusion criteria :<br>≧ 4weeks	Sub-acute and chronic	RMQ : 4.0 (2.1)<br>ODI : 7.8 (3.5)	Forward bending and backward return	Two conditions(15cycle /min):<br>・Symmetric(one target was placed at shoulder height in the anterior sagittal midline so that the target could be reached when standing upright with the arms extended, whereas the second target was placed in the sagittal midline, 50 cm anterior to the knee)<br><br>・Asymmetric (the upper target was moved to the left and the lower target to the right to induce 45 degrees of axial trunk rotation at each target)	30	Magnetic tracking sensors)Liberty, Polhemus, Colchester, VT, USA)	Motion sensors located over T12 and S1	Lumbar – pelvis	Lyapunov analyses	LE	Local stability	No main effect of group on LE<br>No interaction effect of group by symmetry on LE	A Natural Sciences and Engineering Research Council of Canada (NSERC) graduate scholarship during the completion of this study. The authors declare that there is no conflict of interest associated with this work	Between - day reproducibility (ICC(95% C.I) = 0.73(0.22 - 0.92). Refference from other study:<br><br>Graham RB, Sheppard PS, Almosnino S, Stevenson JM. Dynamic spinal stability and kinematic variability across automotive manufacturing work shifts and days. Int J Ind Ergon. 2012;42: 428–434. doi:10.1016/j.ergon.2012.06.001	No
113	Ippersiel	2018	Movement variability in adults with low back pain during sit-to-stand-to-sit	Cross - sectional	n = 37<br>(Sub-acute/Chronic LBP = 16 contol = 21)<br><br>Age and gender :<br>LBP : 5/11(m/f), 30 (±9) years<br>Control : 11/10(m/f), 27(±10) years	LBP:<br>Medical and rehabilitation clinics in Montreal, Quebec, and from the community<br>Unclear regading who conformed diagnosis<br><br>Control:<br>Medical and rehabilitation clinics in Montreal, Quebec, and from the community	Inclusion for LBP<br>・Pain primarily located between the gluteal folds and ribs<br><br>Inclusion for Control<br>N/A<br><br>Exclusion criteria for both group<br>・With no evidence of spinal stenosis or radiculopathy, any serious underlying condition (cauda equina syndrome, cancer, infection), or another specific spinal condition (vertebral compression fracture, ankylosing spondylitis)<br>・Neurological or respiratory conditions that might affect STS, major postural abnormality (e.g. scoliosis of ≥7o), previous spinal surgery or trauma (e.g. spinal fracture), or pregnancy in the past 2 years.	109.9 (±113.5) months<br><br>Inclusion criteria :<br>N/A	Sub-acute and chronic	ODI : 25.3 (7.4)<br>Pain level (NRS) : 3.4 (1.1)<br>STarT Back : 4.4 (1.8)	Sit-to-stand-to-sit	Sitting on a height-adjusted seat, with no back or arm support, such that their thighs were horizontal, their lower legs were vertical, and both feet were flat on the floor. Stand upright, from sitting, and return to sitting, as quickly as possible.	10	An electromagnetic TrakSTAR motion capture system with model 800 sensors (Ascension Technology, Milton, VT, USA)	Thighs, S1, L3, and T12.	Hip - L3S1<br>L3S1 - T12L3	CRP	DP	Coordinative variability	For LHip-L3S1, the LBP group showed significantly more variability in inter-joint coordination (DP) over the full STS movement (P = 0.013; mean difference = −6.95, 95% CI = −12.3 to −1.59)<br><br>When examining separate STS periods, significant group, period, and group- period interaction effects were also found (Table 3), with pairwise comparisons indicating more variability in inter-joint coordination for the LBP group during the start period (P < 0.001; mean differ- ence = −0.325, 95% CI = −0.493 to −0.156)<br><br>For L3S1-T12L3, no between-group difference in DP was found over the full STS movement (P = 0.103). For the STS periods, however, there was a significant period effect, characterized by greater variability over the start and end periods of the STS task, with no group or interaction effects	Quebec Rehabilitation Research Network (REPAR). P. Ippersiel was supported by a Centre for Interdisciplinary Research in Rehabilitation of Greater Montreal (CRIR) doctoral bursary award, and the Richard and Edith Strauss Musculoskeletal Doctoral Fellowship. The funding sources had no involvement in the study or preparation of the manuscript<br><br>Declarations of interest: none	Moderate-excellent intra-rater reliability for lumbar spine and hip(s) coordination analyses during STS. Refference from other study:<br><br>Pourahmadi MR, Ebrahimi Takamjani I, Jaberzadeh S, Sarrafzadeh J, Sanjari MA, Bagheri R, et al. Test-retest reliability of sit-to-stand and stand-to-sit analysis in people with and without chronic non-specific low back pain. Musculoskelet Sci Pract. 2018;35: 95–104. doi:10.1016/j.msksp.2017.11.001	No
32	Mokhtarinia	2016	Trunk coordination in healthy and chronic nonspecific low back pain subjects during repetitive flexion-extension tasks: Effects of movement asymmetry, velocity and load	Cross - sectional	n = 44<br>(Chronic LBP = 22 contol = 22)<br><br>Gender and age :<br>LBP : 22/0 (m/f), 30.2 (±6.1) years<br>Control : 22/0 (m/f), 27.4(±5.1) years	LBP :<br>The university community<br>Medical proffesionals confirmed diagnosis<br><br>Control:<br>The university community	Inclusion for LBP<br>・Had episodic LBP for at least 12 months with limited function due to the back pain<br>・Pain level was lower than two on the 10-point visual-analog scale at the time of the test<br><br>Inclusion for control:<br>・Without history of LBP limiting their function within the previous year<br><br>Exclusion for both groups<br>・Structural deformities of the spine<br>・Pain extending to and/or beyond the gluteal fold<br>・Previous spine, hip or knee surgery<br>・Spinal root pain<br>・Spinal tumors or infections<br>・Neurologic symp- toms such as weakness, tingling or anesthesia in lower extremities<br>・Visual or hearing impairment	≧ 12 months	Chronic	Pain level (VAS) <2	Forward bending and backward return	The task required the subjects to touch a flexion target at their knee height with both hands held together<br><br>Six conditions :<br>・Symmetric/asymetric<br>・high velocity (40cyles per min)/low (20cyles per min)<br>・No-load/loaded (8kg)	30	VICON, Oxford, UK	The C7 and T10 spinous processes, sternum (xiphoid process) and bilaterally on the anterior–superior and posterior-superior iliac spines, lateral femoral epicondyles, and mid-thighs.	Lumbar – pelvis<br>Pelvis - thigh	CRP	DP	Coordinative variability	There was no significant main effect of Group(LBP vs control) for this variable.<br><br>Lumber-pelvic<br>The three-way interaction of Group by Symmetry by Velocity was significant for the lumbar–pelvis coupling<br>-during high velocity conditions, DP was high during asymmetric than symmetric flexion–extension in the control group<br>-during high velocity conditions, DP was same during asymmetric and symmetric flexion–extension in the LBP group<br><br>Pelvis-hip<br>The interaction effect of Group was also significant by Load for DP of the pelvis–thigh coupling<br>-healthy subjects demonstrated greater DP during no-load conditions than during loaded conditions<br>-LBP no difference	N/A	ICC in LBP: 0.64<br>ICC in Control: 0.64	No
87	Moreno	2018	Muscle strength and neuromuscular control in low-back pain: Elite athletes versus general population	Cross - sectional	n = 59<br>(Chronic LBP non-athletes = 15, Chronic LBP athletes = 15 contol non-athletes = 14 contol athletes = 15)<br><br>Gender and age<br>Distribution of ages not clear<br><br>LBP non-athletes : 27.0 (±1.0) years<br>LBP athletes : 23.0 (±2.0) years<br><br>Control non-athletes : 24.0 (±3.0) years<br>Control athletes : 23.0 (±3.0) years	LBP:<br>Not clear<br><br>Control:<br>Not clear	Inclusion for LBP<br>・Having experienced chronic non-specific back pain (i.e., pain not attributable to a recognizable, known specific pathology) within the last 12 weeks<br>・Evidence of LBP induced limitations during daily activities<br><br>Inclusion for Control<br>・Had not experienced lower back pain within the last 12 weeks<br><br>Exclusion for both groups<br>・Any previous history of spinal operation, prolapse, herniated disks, arthritis, mental, neurological, or cardiovascular diseases, sensorimotor deficits, abnormal spinal column structural changes, continuous dependency of pain relieve medication, restrictions from participating in sporting activity from a doctor or undergoing physiotherapist treatment	≧ 12 weeks	Chronic	Pain level(VAS):<br>LBP with non-athletes 3.92(1.7)<br>LBP with athletes 4.54(1.82)	Lifting a box	A pot (1.5 kg) was cyclically moved back and forth between two tables of different heights.The tables were positioned forming an angle of 90◦ to each other, and the participant was standing in the middle of both tables in order to induce 45◦ of axial trunk rotation to each side. The rhythm of 12 cycles/minute (0.20 Hz)	40 cycles	Vicon, Oxford, UK	The anterior and posterior iliac spines and a marker triad (radius 6 mm) was placed at T12 (12th thoracic vertebrae)	Thoracic - hip	Lyapunov analyses	LE	Local stability	No main effect of group on LE(p = 0.375)<br>No interaction effect of group(athlete vs non-athlete) by LBP condition(LBP vs control)	The German Federal Institute of Sport Science on behalf of the Federal Ministry of the Interior of Germany as the major funder.We declare that the results of the study are presented clearly, honestly, and without fabrication, falsification, or inappropriate data manipulation	Not clear	No
127	Pranata	2018	Trunk and lower limb coordination during lifting in people with and without chronic low back pain.	Cross - sectional	n = 72<br>(Chronic LBP with low disability = 25, Chronic LBP with high disability = 18 and Control = 29)<br><br>Age and gender :<br>LBP with low disability : 14/11 (m/f), 42.3 (±11.1) years<br>LBP with moderate - high disability : 6/12 (m/f), 46.7 (±11.8) years<br>Control : 12/17 (m/f), 37.8 (±11.5) years	LBP :<br>Physiotherapy clinic<br><br>Control :<br>The community	Inclusion for LBP with low disability<br>・Reported pain between the level of the twelfth thoracic vertebra (T12) and the gluteal fold<br>・Had persisted for >3 months<br>・ODI < = 20%<br><br>Inclusion for LBP with moderate - high disability<br>・Reported pain between the level of the twelfth thoracic vertebra (T12) and the gluteal fold<br>・Had persisted for >3 months<br>・ODI > 20%<br><br>Exclusion for LBP<br>・Presented with overt neurological signs such as muscle weakness, previous spinal sur- gery, systemic or inflammatory conditions such as rheumatoid arthritis, malignancy, unstable spondylolisthesis<br>・Inability to understand written or spoken English<br><br>Inclusion for Control<br>・Had with no history of choronic LBP	LBP with low disability : 110.2 ± 107.8 months<br><br>LBP with moderate - high disability : 155.2 ± 173.2 months	Chronic	LBP with low disability<br>ODI : 13.2 ± 4.9<br>Pain level (NRS): 3.0 ± 1.6<br><br>LBP with moderate - high disability<br>ODI : 34.4 ± 10.9<br>Pain level (NRS): 4.5 ± 1.9	Lifting a kettlebell	Lifting the kettlebell up to the level of their abdomen using a self-selected pace and technique	2	NaturalPoint, Corvallis, OR	Twenty-one retro-reflective markers of 13 mm diameter were attached to anatomical landmarks of each participant using double sided tape. The thorax, pelvis, thigh and lower leg segments were formed using three retro-reflective markers per segment	Thorax - Pelvis	CRP	DP	Coordinative variability	No difference in DP between groups	A National Health and Medical Research Council R.D. Wright Biomedical Fellowships	all testing variables demonstrated moderate to excellent reliability with ICC's ranging from 0.64 to 0.98	No
21	Shojaei	2017	Timing and magnitude of lumbar spine contribution to trunk forward bending and backward return in patients with acute low back pain	Cross - sectional	n = 38<br>(Acute/sub-acute LBP = 19 contol = 19)<br><br>Gender and age :<br>LBP : 0/19 (m/f), 56 (±9) years<br>Control : 0/19(m/f), 58 (±9) years	Unclear	Inclusion for LBP<br>Not clear<br><br>Inclusion for control<br>・Without any history of LBP or musculoskeletal disorders<br><br>Exclusion for both groups<br>・If they had any significant cognitive impairment, intention to harm themselves or others, or substance abuse	≦ 3 months	Acute/subacute	N/A	Forward bending and backward return	Two conditions<br>- Preferred (bent forward using a preferred pace to reach their maximum trunk rotation, held their maximum trunk rotation for 5 seconds, returned back to the initial upright position, and stood again for 5 seconds)<br><br>- Fast(bent forward as fast as possible to reach their maximum trunk rotation, returned back to the initial upright position, and stood again for 5 seconds)	3	IMU sensor(Xsens Technologies, Enschede, Netherlands)	T10 and the S1 spinous process	Pelvic - thoracic	CRP	DP	Coordinative variability	Significant main effect of group showed DP was smaller in LBP compared to control<br>Forward bend : F = 11.94 p = 0.002<br>Backward bend : F = 4.94 p = 0.034<br><br>No interaction effect : Group(LBP and control), age (40-50, 50-60, 60-70), motion pace (preferred and fast)	No founding	Not clear	No
114	Shojaei	2019	A prospective study of lumbo-pelvic coordination in patients with non-chronic low back pain	Prospective cohort	n = 29<br>(Low - moderate Acute/sub-acute LBP = not clear, Moderate to severe Acute/sub-acute LBP = not clear contol = not clear)<br><br>Age and gender :<br>LBP : 5/24 (m/f)<br>Low-moderate LBP : 55.0 (±10.0) years<br>Moderate-severe LBP : 51.0 (±11.0) years<br><br>Control from an earlier study of their group((Shojaei et al., 2016; Vazirian et al., 2017)	LBP groups :<br>Referred by primary phycian<br><br>Control :<br>Unclear	Inclusion for low - moderate LBP<br>・Pain level < 4 of 10 in all three data collection sessions<br><br>Inclusion for moderate - severe LBP<br>・Pain level > = 4 of 10 in all three data collection sessions<br><br>Exclusion for LBP<br>・Had LBP lasted more than 3 months<br>・Had significant cognitive impairment, intention to harm themselves or others, substance abuse, or did not have access to a telephone<br><br>Exclusion for Control<br>・Any history of LBP during the past year,<br>・Any history of musculoskeletal disorders<br>・Any history of occupational activities that could have substantially influenced the lower back biomechanics	n = not clear<br>(Low - moderate Acute/sub-acute LBP = not clear, Moderate to severe Acute/sub-acute LBP = not clear, contol = not clear)<br><br>Age and gender :<br>LBP : 5/24 (m/f)<br>Low-moderate LBP : not clear<br>Moderate-severe LBP : not clear<br><br>Control from an earlier study of their group((Shojaei et al., 2016; Vazirian et al., 2017)	Acute/subacute(Non-chronic)	Pain level(VAS)<br>Moderate-sever LBP ≧ 4<br>Low-moderate LBP < 4	Forward bending and backward return	Completed two sets of trunk forward bending and backward return tasks at self- selected slow and fast paces in the sagittal plane.<br><br>Two conditions :<br>Preferred(bent forward using a preferred pace to reach their maximum trunk rotation, held their maximum trunk rotation for 5 seconds, returned back to the initial upright position, and stood again for 5 seconds)<br><br>Fast(bent forward as fas as possible to reach their maximum trunk rotation, returned back to the initial upright position, and stood again for 5 seconds)	3	IMU sensor(Xsens Technologies, Enschede, Netherlands)	the T10 and the S1 spinous process	pelvic - thoracic	CRP	DP	Coordinative variability	Low velocity a (self-selected) forward bending :<br>DP of Low-moderate LBP was significant smaller than control.<br>No difference in DP between Moderate-severe LBP and control<br>DP was not differ between patients group at baseline. Significat patients group difference in DP at 3 months(low - moderate LBP: 0.046 ± 0.02 and moderate severe LBP: 0.083 ± 0.03) and 6 months (low - moderate LBP: 0.061 ± 0.04 and moderate severe LBP: 0.098 ± 0.05)<br>Both patient groups DP was larger in 6 months compare to the baseline and 3 month<br><br>High velocity (as fast as possible) forward bending :<br>DP of patients with low-moderate LBP was smaller (>0.018) than that of the other two groups<br>No difference between Moderate-sever LBP and control<br><br>Low velocity backward bending :<br>No difference in DP between groups at baseline.<br>Significat difference in<br><br>High velocity backward bending :<br>No difference in DP between groups	The National Center for Research Resources and the National Center for Advancing Transla- tional Sciences (UL1TR000117) as well as an award (R21OH010195) from the Centers for Disease Control and Prevention.<br><br>We declare that all authors have no financial or personal rela- tionships with other persons or organizations that might inappro- priately influence our work presented therein	Not clear	No
56	Silfies	2009	Trunk control during standing reach: A dynamical system analysis of movement strategies in patients with mechanical low back pain	Cross - sectional	n = 65<br>(Mechnical LBP = 30 contol = 35)<br><br>Gender and age<br>LBP : 19/11 (m/f), 41.1 (±8.9) years<br>Control : 13/12 (m/f) , 38.8 (±9.2) years	LBP:<br>University-based orthopedic surgery practice<br>The spine surgeon confirmed diagnosis<br><br>Control:<br>Not clear	Inclusion for LBP<br>・Low back pain generated by injury or degeneration to spinal region musculoskeletal tissues and not the result of systemic disease. All MLBP subjects had evidence of moderate to severe degenerative disc disease on MRI, as determined by the same spine surgeon.<br><br>Inclusion for Control<br>・No history of low back pain that required attention of a health care provider or that limited function for longer than 3 days<br><br>Exclusion for both groups<br>A history of spinal or hip surgery, osteoporosis, inflammatory joint disease, frank neurolo- gical loss (i.e., lower extremity weakness and sensory loss), pain or paresthesia below the knee, pregnancy, scoliosis, leg length discrepancy or vestibular dysfunction were excluded from the study	3.1 (4) months	Mechnical LBP	RMQ : 8.1 (5.2)<br>Pain level(NRS) : 3.8 (2.2)	Forward bending and backward return	Bilateral forward reach (3s forward bend 3 s backward return) The target distance for each subject was standardized to 50% of their functional reach. Standardizing subjects<br><br>Two conditions :<br>・With and without 4,5 kg load	3	An electromagnetic tracking device (3 Space Fastrak, Polhmeus Inc., Colchester, VT).	The femur (lateral epicondyle), pelvis (S2 spinous process) and lumbar spine (L1 spinous process)	Lumbar – pelvis	CRP	DP	Coordinative variability	Significant main effect of group showed DP was higher in the LBP group during all conditions(F = 5.26, P = 0.03)<br><br>Significant interaction effect of group by movement direction (F = 6.79 p = 0,01). In the LBP group, the DP during backward return was further increase compared to forward bend in both no load and load conditions	No founding	ICC in LBP and Control: 0.64 to 0.82	No

					Population						Task perfomance			Measument			Analysis charactaristics			Results	Others		
ID	Authors	year	Titiles	Study type	Participants(n,age,gender)	Sample source	Eligibility criteria	Symptom duration	Classification (Duration, tyep)	Pain/dasability level/Psychological feature	Task perfomance	Condition	Number of repetition,time	Measurement Apparatus	Sensor/maker placements	Angle definition	Analysis technique	Analysis metric	Type of variability	Main results	Founding source	Reliable data acquisition	Power calculation
52	Williams	2013	A novel approach to the clinical evaluation of differential kinematics of the lumbar spine	Cross - sectional	n = 28<br>(Acute LBP = 16 Chronic LBP = 12)<br><br>Gender and age :<br>Acute LBP : 10/6 (m/f), 42.6 (±7.2) years<br>Chronic LBP : 7/5 (m/f) , 34.5 (±10.0) years	LBP :<br>General practitioner referrals to local therapy departments.	Inclusion for LBP<br>・Pain confined to between the lower ribs and inferior gluteal folds<br>・18-55 years old<br>・Seeking healthcare for their LBP and evoked pain on at least 3 of the test movements<br>・Acute pain was pain present for less than three weeks and chronic defined as pain present on at least three days per week for greater than twelve months<br><br>Exclusion for LBP<br>・A history of tumours, spinal fractures, surgery, rheumatological or neurological diseases<br>・Any neurological signs or symptoms	Acute LBP : 12.0 ±┐7.3 days<br>Chronic LBP : 4030.2 ± 2992.2 days	Acute<br>Chronic	VAS :<br>Acute LBP : 6.15 ±┐1.89<br>Chronic LBP : 4.57 ±┐2.21<br><br>TSK :<br>Acute LBP : 39.3 ±┐4.1<br>Chronic LBP : 38.3 ±┐7.5	Forward bend<br>Backward bend<br>Left side bending<br>Right side bending<br>Left rotation<br>Right rotation<br>Box lift	Standing upright and completed three trials of flexion, extension, left side bending, right side bending, left rotation, right rotation and a box lift. The box (460 │ 260 │ 300 mm) was posi- tioned on markers ensuring identical placement and weighed 3 kg	3	IMU sensor(3DM-GX3-25, Microstrain, VT, USA)	S1 and L1 spinouys process	Lumbar–pelvis	The RMS difference between the actual data and the polynomial of angular velocity-ROM plots.The beginning and end of the polynomial was fixed to match the collected data	RMS	Movement irreqularity	Significant between group difference in motion irregularity values during extension, left and right side flexion, left rotation and lifting.The quartile with the greatest irregularity was consistently the second for all movements except left rotation and lifting in the ChronicLBP group	N/A	The mean CMC values were found to be good across all repeated range of motion curves for the ALBP (0.83e0.97 ┐ 0.02e0.09) and CLBP (0.72e0.94 ┐ 0.03e0.26) groups. This was also seen for angular velocity curves (0.83e0.94 ┐ 0.04e0.10 ALBP and 0.71e 0.91 ┐ 0.05e0.18 CLBP).<br>The mean CMC values for angular acceleration curves show moderate consistency across the different movements tested (0.61 e0.77 ┐ 0.10e0.18 ALBP and 0.52e0.73 ┐ 0.10e0.26 CLBP). The RMSE magnitudes across all kinematic variables were small for all repeated movements<br>Mean ICC values were shown to be good across all movements for both groups (0.71e0.99) and mean absolute differences of repeated peak measures were small, <3.7¶ and <5.6¶s┘1 for ROM and angular velocity and moderate <13.5¶s┘2 for angular acceler- ation<br>The similarity in irregularity scores between repeated trials was moderate-to-good for all the movements investigated (0.49e0.86 ALBP and 0.50e0.83 CLBP group). Mean absolute differences between repeated movements were small (1.2e5.5) suggesting good reliability of such a method	No
146	Williams	2014	An experimental study investigating the effect of pain relief from oral analgesia on lumbar range of motion, velocity, acceleration and movement irregularity	Intervention<br><br>oral analgesia : self-administered between pre and post movement trilas	n = 40<br>(Acute LBP = 20, Chronic LBP = 20)<br><br>Age and gender :<br>Acute LBP : 11/9 (m/f), 42.7 (±6.8) years<br>Chronic LBP : 11/9 (m/f), 36.6 (±10.8) years/	LBP groups<br>Local physiotherapy and chiropractic clinics	Inclusion for LBP<br>・Required to report pain during at least three of the tested movements<br>・Pain confined to between lower ribs and inferior gluteal folds<br>・Aged 18–55 years old<br>・Seeking healthcare for LBP<br>・Acute – pain present for less than 3 weeks on a history of no pain for at least 12 months<br>・Chronic – pain present on at least 3 days per week for at least 52 weeks<br><br>Exclusion for LBP<br>・Had history of tumors, spinal fractures, surgery, neurological signs or symptoms, rhuematological or Neurological disease, known spinal deformity	Acute LBP : 12.3 ± 6.7 days<br>Chronic LBP : 9.4 ± 7.4 years	Mechanical Acute and Chronic and	VAS<br>Acute 6.22 ± 16.6<br>Chronic 4.6 ± 2.2<br><br>TSK :<br>Acute 39.0 ± 4.8<br>Chronic 38.9 ± 6.9	Forward and backward return, side-bending, twisting and lifting	No constraints were placed on any of the movements. This ensures the movements were completed naturally, better reflects the clinical situ- ation and mirrors functional tasks. The object to be lifted was a box with dimensions 460 × 260 × 300 mm which weighed 3 kg. The magnitude of the worst pain evoked by the movements was measured using a visual analogue scale completed following each of the three movement trials.	3	IMU sensor (GX3-25, Microstrain, VT, USA)	The L1 and S1	Lumbar–pelvis	The RMS difference between the actual data and the polynomial of angular velocity-ROM plots.The beginning and end of the polynomial was fixed to match the collected data	RMS	Movement irreqularity	Pain reduction had no effect on movement irregularity for each quartile of any movement tested, with the exception of quartile 3(maximum angular displacement to minimum angular velocity) for flexion in the ALBP group(decrease in movement irregularity), and quartile 3 for side bending in the Chronic LBP group(increase in movement irregularity)		Not clear	Yes

LBP: low back pain; VAS; Visual analog scale; NRS; Numerical rating scale; ODI: Oswestry Disability Index; RMQ: Roland Morris Disability Questionnaire; TSK: tampa scale of kinesiophobia; ROM: Range of Motion; DP: Deviation Phase; CRP: continuous relative phase; RQA: Recurrence quantification analysis; GEM: Goal Equivalent Manifold; LE: Lyapunov Exponents; RMS: Root Mean Square; SD: Standard Deviation; CV: Coefficient of Variation; VR: Variance Ratio; DET: determinism; REC:Recurrence rate; SaEn: Sample Entropy; FM: Floquet multipliers; NME: Neuromuscular Exercises; RPT: Random-Perturbation Therapy
